# Effectiveness of a web-enabled psychoeducational resource for postpartum depression and anxiety among women in British Columbia

**DOI:** 10.1007/s00737-024-01468-8

**Published:** 2024-05-06

**Authors:** Claire G Lawrence, Genevieve Breau, Lily Yang, Orli S Hellerstein, Catriona Hippman, Andrea L Kennedy, Deirdre Ryan, Barbara Shulman, Lori A Brotto

**Affiliations:** 1 Women’s Health Research Institute, BC Women’s Hospital + Health Centre, Vancouver, BC Canada; 2 Department of Obstetrics & Gynaecology, Faculty of Medicine, University of British Columbia, Vancouver, BC Canada; 3 School of Human Sciences, Faculty of Education, Health, and Human Sciences, University of Greenwich, Old Royal Naval College, Park Row, London, SE10 9LS UK; 4 Faculty of Medicine, University of British Columbia, Vancouver, BC Canada; 5 Department of Psychology, University of British Columbia, Vancouver, BC Canada; 6 Faculty of Nursing, University of Calgary, Calgary, AB Canada; 7 Reproductive Mental Health Program, BC Women’s Hospital + Health Centre, Vancouver, BC Canada; 8 Department of Psychiatry, Faculty of Medicine, University of British Columbia, Vancouver, BC Canada

**Keywords:** Postpartum depression, Postpartum anxiety, eHealth, Psychoeducation, Digital Health

## Abstract

**Purpose:**

Postpartum depression (PPD) and anxiety (PPA) affect nearly one-quarter (23%) of women in Canada. eHealth is a promising solution for increasing access to postpartum mental healthcare. However, a user-centered approach is not routinely taken in the development of web-enabled resources, leaving postpartum women out of critical decision-making processes. This study aimed to evaluate the effectiveness, usability, and user satisfaction of PostpartumCare.ca, a web-enabled psychoeducational resource for PPD and PPA, created in partnership with postpartum women in British Columbia.

**Methods:**

Participants were randomized to either an intervention group (*n* = 52) receiving access to PostpartumCare.ca for four weeks, or to a waitlist control group (*n* = 51). Measures evaluating PPD (Edinburgh Postnatal Depression Scale) and PPA symptoms (Perinatal Anxiety Screening Scale) were completed at baseline, after four weeks, and after a two-week follow-up. User ratings of website usability and satisfaction and website metrics were also collected.

**Results:**

PPD and PPA symptoms were significantly reduced for the intervention group only after four weeks, with improvements maintained after a two-week follow-up, corresponding with small-to-medium effect sizes (PPD: partial η^2^ = 0.03; PPA: partial η^2^ = 0.04). Intervention participants were also more likely than waitlist controls to recover from clinical levels of PPD symptoms (χ ^*2*^ (1, *n* = 63) = 4.58, *p* = .032) and PostpartumCare.ca’s usability and satisfaction were rated favourably overall.

**Conclusion:**

Findings suggest that a web-enabled psychoeducational resource, created in collaboration with patient partners, can effectively reduce PPD and PPA symptoms, supporting its potential use as a low-barrier option for postpartum women.

**Trial Registration:**

Protocol for this trial was preregistered on NIH U.S. National Library of Medicine, ClinicalTrials.gov as of May 2022 (ID No. NCT05382884).

## Introduction

Postpartum Depression (PPD) is a leading cause of maternal morbidity and mortality (Oates 2003). In 2019, Statistics Canada reported that PPD symptoms affect 10% of Canadian women, while an additional 8% experience comorbid PPD and postpartum anxiety (PPA) symptoms, and another 5% experience symptoms of PPA only (Government of Canada 2019).

Untreated PPD and PPA can severely impact a parent’s mental and physical health, offspring cognitive and motor development, and the overall wellbeing of a family unit (Agnafors et al. 2013; Brummelte and Galea 2016; Fitelson et al. 2010). Given the impacts of postpartum mental illness, effective strategies for prevention, management, and treatment are critical (Zhou et al. 2022).

Current best practice guidelines for perinatal mental healthcare within British Columbia (BC) recommend treatment based on symptom severity (Williams et al. 2014) with nonpharmacological interventions (e.g., psychotherapy, psychoeducation) being recommended as first-line options before considering pharmacological treatment for mild-to-moderate cases, and combination (psychological and pharmacological) in more severe cases (Cuijpers et al. 2023; Donker et al. 2009; Williams et al. 2014).

Despite these existing effective options, barriers reduce access to these supports (Button et al. 2017; Jones 2019). Key barriers impacting help-seeking include social stigma, financial constraints, insufficient education and awareness, and logistical issues with attending in-person appointments (Button et al. 2017; Jones 2019).

eHealth involves the translation of health interventions from traditional in-person provision to delivery via telephone, mobile application, or web-enabled platforms and is a promising solution for increasing access to mental healthcare (Enam et al. 2018; Eysenbach 2001; Zhao et al. 2021). Previously, eHealth interventions have been developed and assessed to effectively reduce symptoms of PPD and PPA (Lee et al. 2016; Milgrom et al. 2016; Ngai et al. 2015; O’Mahen et al. 2014; Pugh et al. 2016; Wozney et al. 2017; Zhao et al. 2021).

A user-centered design approach involves an iterative process of incorporating end-user input at each stage of eHealth intervention development (Still and Crane 2017; User-Centered Design Basics 2017). This approach establishes the importance of understanding the identity and voice of end-users and involving them early on and regularly to design relevant and valuable resources (Still and Crane 2017; User-Centered Design Basics 2017). According to the standards for evaluating eHealth interventions, the conceptualization and design phases are key points of evaluation (Eng 1999), yet many eHealth interventions do not involve patient partners’ voices throughout, and instead only include them at the end of the study (Enam et al. 2018).

Although existing eHealth interventions for postpartum mental health have been successful in reducing symptoms of mental illness, few have taken a true user-centered approach by involving patient voices in the conceptualization and design of these platforms. For example, O’Mahen et al. (2014) utilized feedback from a qualitative study assessing women’s preferences for a perinatal CBT program when developing content for an internet behavioural-activation-based treatment for PPD, However, this feedback was not specific to the delivery of this program via eHealth and, therefore, user preferences associated with using technology for the delivery of the intervention may have been missed. As well, Pugh et al. (2016) evaluated the efficacy of internet-based CBT program for PPD and assessed qualitative feedback from users of their program and identified areas for improvement, but the intervention itself appears to have been conceptualized and designed by researchers only, without input from the intended user group, PPD patients. To address this gap in the literature, there is a need for research that follows a user-centered approach by involving patient partners in the initial eHealth development processes to create an intervention that best addresses the needs, values, and preferences of those facing postpartum mental illness.

This study is the third phase of a research project with the goal of developing an effective and accessible web-enabled psychoeducational resource for individuals in BC, Canada, experiencing postpartum mental health concerns. Phase One identified the unmet needs of those experiencing postpartum mental illness and investigated how a web-enabled intervention could meet these needs, including specific content and features desired (Lackie et al. 2021). Lackie et al. (2021) pointed out that many of the existing eHealth interventions for postpartum mental health relied primarily on delivering psychotherapy via technology (e.g., delivering Cognitive Behavioural Therapy (CBT) or Behavioural Activation adapted to online platforms) which may not address some of the key unmet needs of women identified within the study such as the provision of education using evidence-based information to address the lack of knowledge surrounding perinatal mental illness, a resource hub for accessing relevant support services, and information on self-care and peer support. Therefore, authors concluded that a web-enabled psychoeducational intervention would be best suited to address the unmet needs of women in BC.

Phase Two designed the psychoeducational web-enabled resource for postpartum mental health, PostpartumCare.ca, guided by a multidisciplinary advisory committee of patient partners, mental health clinicians, researchers, and community organization partners with firsthand expertise addressing perinatal mental illness. Phase Two also involved a feasibility study to collect user feedback regarding visual layout, content, and overall website satisfaction (Siddhpuria et al. 2022). This feedback was incorporated in the website design, guided by the web development advisory committee.

The objective of the current Phase Three randomized controlled trial was to evaluate the effectiveness, usability, and user satisfaction of PostpartumCare.ca. Effectiveness was evaluated using primary outcomes of PPD and PPA symptoms. Implementation outcomes included user ratings of website usability and satisfaction and website metrics collected to gain an understanding of participant usage patterns. We hypothesized that: (1) following a four-week usage period of PostpartumCare.ca, intervention participants would see a greater reduction in depression and anxiety symptoms compared to waitlist control participants and that this would be maintained after a two-week follow-up period. (2) We also expected that PostpartumCare.ca would be rated favourably by intervention participants according to implementation outcome measures of usability and satisfaction.

## Materials and methods

### Inclusion/exclusion criteria

Participants were required to be 19 years of age or older, have given birth within the past 12 months, reside in BC, have occasional or regular access to the internet (via computer, smartphone, tablet, etc.), be able to communicate in English (i.e., be able to read, write, and speak conversational English), and be experiencing symptoms of postpartum depression and/or anxiety as per the EPDS for depression (receive a score ≥ 9), and the Perinatal Anxiety Screening Scale (PASS) for anxiety (receive a score ≥ 26) (Cox et al. 1987; Somerville et al. 2014).

### Recruitment

Protocol for this trial was preregistered on NIH U.S. National Library of Medicine, ClinicalTrials.gov as of May 2022 (ID No. NCT05382884). Research Ethics approval for this study was obtained from the University of British Columbia and the BC Children’s and Women’s Hospital Research Ethics Board (REB number: H21-01379)). Participants were recruited primarily online, through advertisements posted on social media pages.

### Power and sample size

Sample size was determined for a two-way mixed ANOVA with two groups and three times of assessment using G*Power software (version 3.1) with a goal of detecting a small-to-medium effect using a significance level α = 0.05 and a minimum power of 80%. With these parameters, the recommended total sample size was *N* = 74. Considering an expected attrition rate of 20%, we aimed to enroll a minimum of 93 participants.

### Procedures

Data were collected from June 2022 through April 2023. Enrolled participants were randomly allocated to either the intervention or waitlist control group by simple randomization using a random number generator (0 – *waitlist control*, 1 – *intervention*). Participants were not notified of their group allocation, instead they were only notified via email of when their start date would be for website access.

Each arm of the study was six weeks in length and participants in both groups completed online questionnaires evaluating PPD and PPA symptoms at three time points: (T1) at baseline, prior to the intervention/waitlist control period; (T2) after a four-week intervention/waitlist control period; and (T3) after a two-week follow-up period. At T2, intervention participants completed an additional questionnaire assessing website usability and satisfaction and their website metrics were collected. In cases where five consecutive unsuccessful contact attempts were made to remind participants to complete questionnaires, the participant was deemed lost to follow-up.

### Description of the intervention/waitlist control conditions

Participants in the intervention group received access to PostpartumCare.ca immediately following the completion of T1 questionnaires for a period of four weeks. Waitlist control participants received website access after T3, following all data collection.

**Fig. 1:**
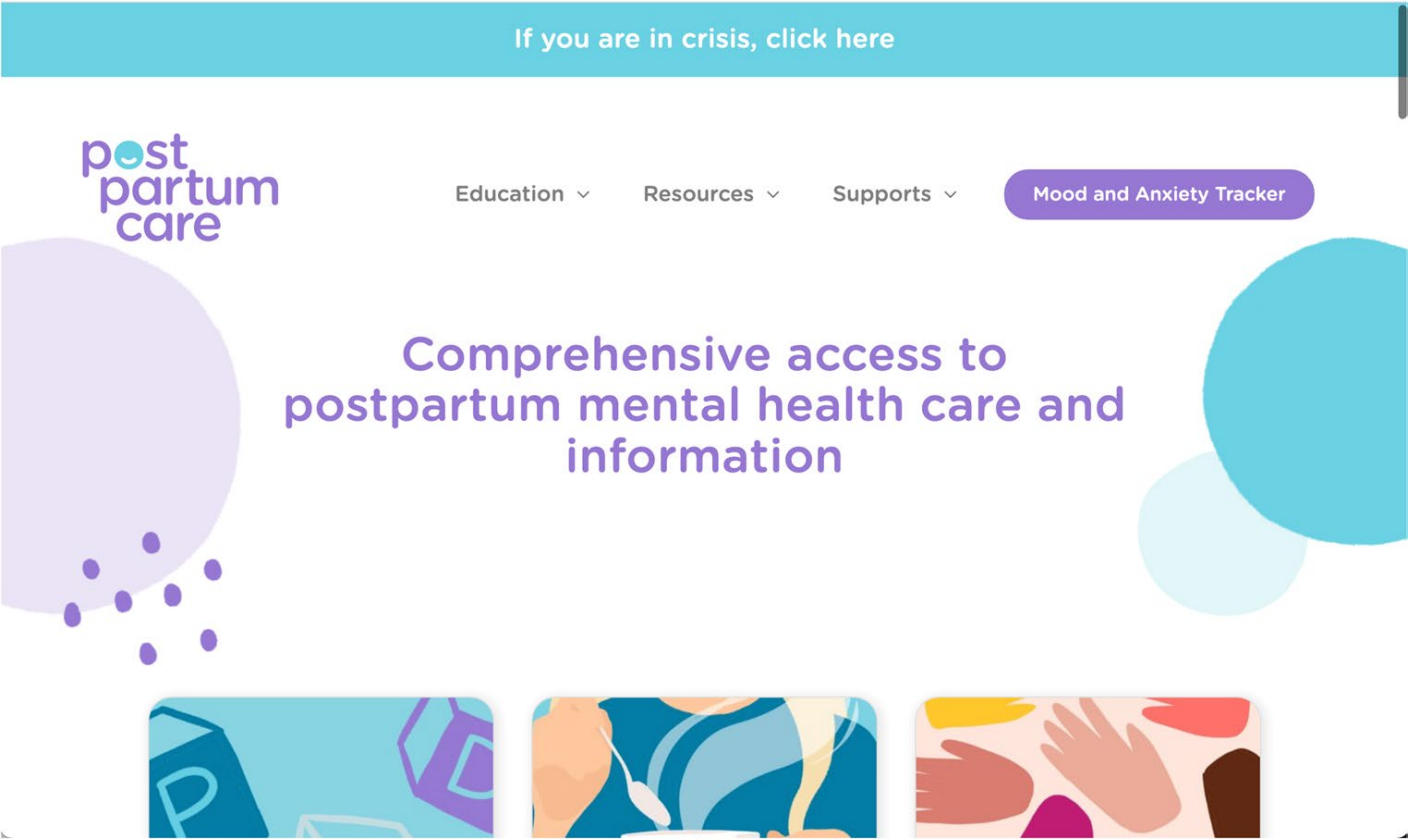
Desktop interface of postpartumCare.ca

PostpartumCare.ca is composed of a main landing page containing tabs for three website sections: Education, Resources, and Supports, as well as a separate Mood/Anxiety Tracker section (Table 1; Fig. 1). The Education, Resources, and Supports sections contain didactic text related to PPD and PPA, links to local BC and remote (online/phone) support services, and information on social supports and self-care practices, respectively. The Mood/Anxiety Tracker section permits users to sign in and input mood and/or anxiety symptoms to track symptom changes over time. Users can select either a “Mood” or “Anxiety” tab and are prompted to rate their mood (i.e., how you feel most of the day, over the past week) on a sliding scale from 0 to 10, with higher scores indicating better mood, and/or their anxiety symptoms (i.e., how anxious you feel most of the day, over the past week) on a sliding scale from 0 to 10, with higher scores indicating less anxiety. For each entry, users are also given options to click on specific symptoms experienced (e.g., losing interest in favourite activities, losing ability to concentrate, feeling guilty or worthless, etc.) and/or write an extra note in an open textbox before submitting their entry. Once submitted, users can click on a “Graphs” tab to see their mood/anxiety ratings plotted over time (e.g., over 3 days, 7 days, 14 days, or 30 days) or on a “Resources” tab for links to resources provided based on specific symptoms entered. All website sections also contained links to outsources (e.g., other websites/resources) relevant to the material within each section.

**Table 1:** Description of website content by section

Website Section	Content Description	
*Education*	• Introduction to perinatal mental health, definitions of PPD/PPA, differences between normal and serious symptoms, and types of perinatal anxiety disorders • Information on causes and protective factors for perinatal mental illness (e.g., biological and psychosocial risk factors; added risk factors for Indigenous populations, partners, and newcomers to Canada) • Information on screening and diagnosis of perinatal mental illness (e.g., who to see for diagnosis, who should be screened, how screening works), and descriptions of treatment and management options (e.g., psychotherapy, psychoeducation, medication, mindfulness, etc.) • Additional considerations including information on perinatal mental health and pregnancy loss/stillbirth, surrogacy, LGBTQIA2S + populations, cultural impacts and traditional practices, adoptive parents, and differences of grief vs. depression • Frequently asked questions regarding perinatal mental health (e.g., “What does perinatal mean?”, “Can I recover from PPD?”, “I don’t want to take medications, are there alternative options?”, etc.)	
*Supports*	• Information on self-care practices, accessing/ building social networks and community groups, and connecting with friends and family • *Shared stories of four women’s experiences with PPD/PPA	
*Resources*	• Links to in-person and remote (online/phone) resources for perinatal mental health based on geographic location within BC • Map feature with BC-based resources pinned based on geographic location • Specific resources for general perinatal mental health support, crisis services, province-wide resources, resources that require referral by a physician, and resources for partners	
*Mood/Anxiety Tracker*	• Separate sign-in page to access mood/anxiety tracking feature • Mood Tracker is comprised of three entry options: (1) option to enter mood rating on a sliding scale from 0 to 10, with higher scores indicating better mood; (2) option to click on specific symptoms experienced; (3) option to enter a note relating to mood in textbox • Anxiety Tracker is also comprised of three entry options: (1) option to enter anxiety rating on a sliding scale from 1 to 10, with higher scores indicating less anxiety; (2) option to click on specific symptoms experienced; (3) option to enter a note relating to anxiety in textbox • Links to resources are provided based on entry of specific symptoms experienced once an entry is submitted • Graph is available to visualize tracked mood/anxiety ratings over time (e.g., over 3 days, 7 days, 14 days, or 30 days)	

*Note* BC = British Columbia; LGBTQIA2S + = Lesbian, Gay, Bisexual, Transgender, Queer and/or Questioning, Intersex, Asexual and/or Aromantic and/or Agender, Two-Spirit + (using + at the end of the acronym aims to acknowledge that many sexually and gender diverse people don’t identify with the terms included in the acronym); PPA = Postpartum Anxiety; PPD = Postpartum Depression

*Stories were shared anonymously by four women who took place in previous focus group research

PostpartumCare.ca was made to be accessible on any computer, tablet, or smartphone device to allow for use on any personal devices (e.g., smartphones or laptops) or public devices (e.g., library computers). Prior to receiving access, intervention participants were notified of all website sections, given a brief description of their contents, and given four weeks to use the website as often/in whatever capacity desired with the knowledge that they would be asked to complete online questionnaires evaluating their experience using the website after this four-week period. No additional instructions or reminders for website use were given. Having participants use the website as desired (i.e., without specific time and/or frequency requirements) was intended to reflect what realistic use of the website might be for BC’s postpartum population in accordance with principles of effectiveness/pragmatic trials (Kim 2013).

### Measures

#### Demographics

Demographic information was obtained through an investigator-derived self-report questionnaire (see Table 2 for demographic variables collected).

**Table 2:** *Demographic characteristics of intervention and waitlist control group participants at baseline (T1)*

Measure	Intervention	Waitlist control	Total
Number of Participants, *N*	52	51	103
*Age (years), *M ± SD*	31.60 ± 5.11	34.25 ± 4.84	32.91 ± 5.13
Gender, *n* (%)			
Woman	52 (100)	51 (100)	103 (100)
Relationship status, *n* (%)			
Unmarried without a partner	2 (3.8)	1 (2.0)	3 (2.9)
Unmarried with a partner	5 (9.6)	2 (3.9)	7 (6.8)
Married	37 (71.2)	39 (76.5)	76 (73.8)
Common law	7 (13.5)	9 (17.6)	16 (15.5)
Divorced	1 (1.9)	0 (0)	1 (1.0)
Ethnicity, *n* (%)			
Arab/West Asian	1 (1.9)	3 (5.9)	4 (3.9)
Black	4 (7.7)	1 (2.0)	5 (4.9)
Chinese	6 (11.5)	7 (13.7)	13 (12.6)
Filipino	0 (0)	3 (5.9)	3 (2.9)
Hispanic/Latin American	1 (1.9)	4 (7.8)	5 (4.9)
Korean	1 (1.9)	0 (0)	1 (1.0)
South Asian	1 (1.9)	1 (2.0)	2 (1.9)
Southeast Asian	1 (1.9)	2 (3.9)	3 (2.9)
White	35 (67.3)	31 (60.8)	66 (64.1)
Other	6 (11.5)	4 (7.8)	10 (9.7)
Prefer not to answer	1 (1.9)	1 (2.0)	2 (1.9)
Indigenous identity, *n* (%)			
Yes	4 (7.7)	6 (11.8)	10 (9.7)
No	48 (92.3)	45 (88.2)	93 (90.3)
Type of Indigenous identity, *n* (%)			
First Nations	2 (3.8)	3 (5.9)	5 (4.9)
Metis	1 (1.9)	3 (5.9)	4 (3.9)
Cree	1 (1.9)	0 (0)	1 (1.0)
Sexual orientation, *n* (%)			
Asexual	2 (3.8)	4 (7.8)	6 (5.8)
Bisexual	5 (9.6)	4 (7.8)	9 (8.7)
Heterosexual	41 (78.8)	38 (74.5)	79 (76.7)
Pansexual	1 (1.9)	2 (3.9)	3 (2.9)
Prefer not to answer	3 (5.8)	3 (5.9)	6 (5.8)
Annual household income (CAD), *n* (%)			
Less than $20,000	2 (3.8)	1 (2.0)	3 (2.9)
$20,000-$39,999	4 (7.7)	2 (3.9)	6 (5.8)
$40,000-$59,999	3 (5.8)	4 (7.8)	7 (6.8)
$60,000-$79,999	5 (9.6)	5 (9.8)	10 (9.7)
$80,000-$99,999	9 (17.3)	5 (9.8)	14 (13.6)
$100,000-$119,999	4 (7.7)	10 (10.6)	14 (13.6)
$120,000-$139,999	6 (11.5)	6 (11.8)	12 (11.7)
$140,000-$159,999	8 (15.4)	3 (5.9)	11 (10.7)
More than $160,000	6 (11.5)	9 (17.6)	15 (14.6)
Prefer not to answer	5 (9.6)	6 (11.8)	11 (10.7)
Employment status, *n* (%)			
Full time	3 (5.8)	3 (5.9)	6 (5.8)
Part time	1 (1.9)	1 (2.0)	2 (1.9)
On disability	3 (5.8)	0 (0)	3 (2.9)
On maternity leave	41 (78.8)	39 (76.5)	80 (77.7)
Self-employed	0 (0)	1 (2.0)	1 (1.0)
Stay-at-home caregiver	2 (3.8)	4 (7.8)	6 (5.8)
Unemployed	0 (0)	2 (3.9)	2 (1.9)
Other	2 (3.8)	1 (2.0)	3 (2.9)
*Years of school, *M ± SD*	16.06 ± 3.16	17.64 ± 2.83	16.86 ± 3.08
*Highest education level, *n* (%)			
Attended some high school	2 (3.8)	1 (2.0)	3 (2.9)
Graduated high school or earned GED	3 (5.8)	1 (2.0)	4 (3.9)
Attended some college/university	11 (21.2)	9 (17.6)	20 (19.4)
Graduated 2-year college/university	8 (15.4)	2 (3.9)	10 (9.7)
Graduated 4-year college/university	21 (40.4)	17 (33.3)	38 (36.9)
Post-Graduate degree	7 (13.5)	20 (39.2)	27 (26.2)
Prefer not to answer	0 (0)	1 (2.0)	1 (1.0)
^a^Region of residence within BC, *n* (%)			
Interior BC	4 (7.7)	8 (15.7)	12 (11.7)
Northern BC	6 (11.5)	5 (9.8)	11 (10.7)
Lower Mainland/Fraser Valley (not including Vancouver)	18 (34.6)	13 (25.5)	31 (30.1)
Vancouver	12 (23.1)	9 (17.6)	21 (20.4)
Vancouver Island	12 (23.1)	16 (31.4)	28 (27.2)
Number of people living in household, *M ± SD*	3.88 ± 1.22	3.86 ± 1.11	3.87 ± 1.16
Number of adults (age 18 or older) living in household, *M ± SD*	2.25 ± 1.06	2.14 ± 0.72	2.19 ± 0.91
Number of children, *M ± SD*	1.67 ± 1.08	1.71 ± 0.81	1.69 ± 0.95
Ages of children, *n* (%)			
At least 1 child under 6 years of age	52 (100)	51 (100)	103 (100)
At least 1 child between 6 and 12 years of age	4 (7.7)	6 (11.8)	10 (9.7)
At least 1 child between 13 and 18 years of age	4 (7.7)	3 (5.9)	7 (6.8)
Number of pregnancies, *M* ± *SD*	2.44 ± 2.05	2.34 ± 1.26	2.39 ± 1.69
Number of live births, *M ± SD*	1.62 ± 1.05	1.63 ± 0.73	1.62 ± 0.90
Number of still births, *M ± SD*	0.04 ± 0.19	0.06 ± 0.24	0.05 ± 0.22
Number of infant losses, *M ± SD*	0.00 ± 0.00	0.02 ± 0.14	0.01 ± 0.10
Number of abortions, *M ± SD*	0.17 ± 0.47	0.26 ± 0.52	0.21 ± 0.50
Number of miscarriages, *M ± SD*	0.67 ± 1.42	0.51 ± 0.76	0.60 ± 1.14
Pregnancy type (of most recent pregnancy), *n* (%)			
Singleton pregnancy	51 (98.1)	49 (96.1)	100 (97.1)
Multiple pregnancy	1 (1.9)	2 (3.9)	3 (2.9)
Delivery type (of most recent birth), *n* (%)			
Vaginal delivery	30 (57.7)	27 (52.9)	57 (55.3)
Planned caesarian section	8 (15.4)	12 (23.5)	20 (19.4)
Emergency caesarian section	14 (26.9)	12 (23.5)	26 (25.2)
Ability to receive pregnancy, delivery, and/or postpartum care within the community of residence, *n* (%)			
Yes	42 (80.8)	39 (76.5)	81 (78.6)
No	10 (19.2)	12 (23.5)	22 (21.4)
Delivery location (of most recent birth), *n* (%)			
At hospital	49 (94.2)	49 (96.1)	98 (95.1)
At home	3 (5.8)	2 (3.9)	5 (4.9)
PPD diagnosis by a health professional, *n* (%)			
Yes	11 (21.2)	16 (31.4)	27 (26.2)
No	41 (78.8)	35 (68.6)	76 (73.8)
^b^Number of PPD diagnoses, *M ± SD*	1.27 ± 0.47	1.31 ± 0.60	1.30 ± 0.54
PPA diagnosis by a health professional, *n* (%)			
Yes	11 (21.2)	14 (28.0)	25 (24.3)
No	41 (78.8)	36 (72.0)	77 (74.8)
^b^Number of PPA diagnoses, *M ± SD*	1.64 ± 1.21	1.14 ± 0.36	1.36 ± 0.86
^a^Medication use to treat mental health conditions such as PPD/PPA (i.e., SSRI, NDRI), *n* (%)			
Yes	15 (28.8)	12 (23.5)	27 (26.2)
No	37 (71.2)	39 (76.5)	76 (73.8)

*Note* BC = British Columbia; CAD = Canadian Dollar; GED = General Educational Development; NDRI = Norepinephrine and Dopamine Reuptake Inhibitor; PPA = Postpartum Anxiety; PPD = Postpartum Depression; SSRI = Selective Serotonin Reuptake Inhibitor

*Significant difference between groups *p* < .05

^a^Variable was entered as a “fill in the blank” option on the demographic questionnaire. As such, it was recoded as a categorical variable to calculate descriptive statistics

^b^Means and standard deviations for number of PPD/PPA diagnoses are reported for those who answered “yes” to being previously diagnosed (*n* = 27 for PPD; *n* = 25 for PPA)

#### PPD symptoms

PPD symptoms were measured using the Edinburgh Postnatal Depression Scale (EPDS), a reliable and valid 10-item self-report measure used internationally to evaluate PPD symptoms (Cox et al. 1987). To avoid false negatives and include participants who might meet PPD diagnostic criteria (i.e., scores indicating possible depression), an EPDS score ≥ 9 was used as an inclusion criterion (Levis et al. 2020; Williams et al. 2014). Cronbach’s alpha for the present sample was high (α = 0.85) (DeVellis 2003; Kline 2005).

#### PPA symptoms

PPA symptoms were measured using the Perinatal Anxiety Screening Scale (PASS), a reliable and valid 31-item self-report measure for anxiety screening in perinatal populations (Somerville et al. 2014). The recommended cut-off score of ≥ 26 for differentiating high and low risk of presenting with an anxiety disorder was used as an inclusion criterion (Somerville et al. 2014). Cronbach’s alpha for the present sample was high (α = 0.94) (DeVellis 2003; Kline 2005).

*Website Usability and Satisfaction*: A brief three-question Patient Global Impression of Change (PGIC) questionnaire was adapted for this study based on the measure developed by Hurst and Bolton (2004) to assess (1) perceived improvement of PPD and/or PPA symptoms; (2) perceived improvements in overall quality of life; and (3) overall satisfaction towards the intervention. The System Usability Scale (SUS) was used to evaluate usability parameters such as website ease of use, complexity, and function integration (Brooke 1995). The Patient Education Materials Assessment Tool (PEMAT) was used to evaluate the understandability and actionability of website patient education materials (Shoemaker et al. 2014). The User-Perceived Web Quality Instrument (UPWQI) was used to measure four dimensions of perceived web quality: technical adequacy, content quality, specific content, and appearance (Aladwani and Palvia 2002).

#### Website metrics

Website metrics were collected using Matomo Analytics to describe how participants used PostpartumCare.ca and included average number of page visits per participant, average time spent visiting each section per session (in minutes), total number of pageviews, and most visited outsources per website section. Data were available for the entire sample (not individual).

### Data analysis

Data were analyzed following intention-to-treat (ITT) principles. Independent *t*-tests were conducted for all continuous variables and chi-square tests were conducted for all categorical variables to compare the randomized groups at baseline. To evaluate primary outcomes, two-way mixed ANOVAs with one between-subject factor (group: intervention, waitlist control) and one within-subject factor (time of assessment: T1, T2, T3) were conducted. Results of participant demographic questionnaires and implementation outcome measures were reported descriptively.

### Calculating clinical significance

Clinically significant improvement was assessed for both primary outcomes. For PPD, clinically significant change on the EPDS was calculated according to procedures outlined by Matthey (2004). Participants who saw both a reliable score decrease of four or more points and cut-off change from > 12 to ≤ 12 from T1 to T3 were classified as “recovered” from PPD (Matthey 2004). Clinically significant improvement for PPA was defined as a change in PASS total scores from ≥ 26 to < 26 from T1 to T3 (Somerville et al. 2014). Chi-square tests were conducted to determine if there was an association between group and clinically significant improvement for PPD and PPA.

## Results

### Participants

**Fig. 2:**
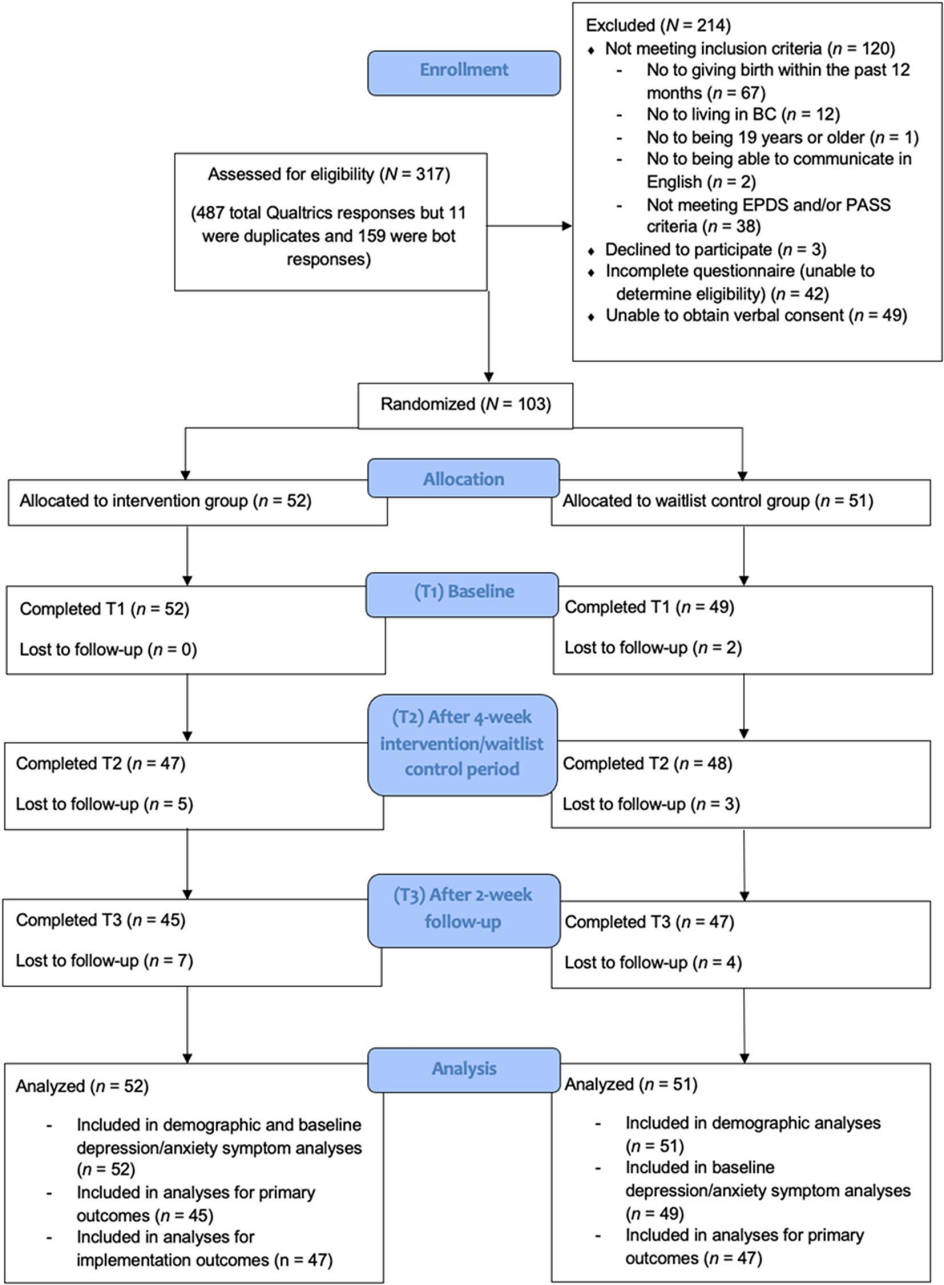
Consort diagram

**Fig. 3:**
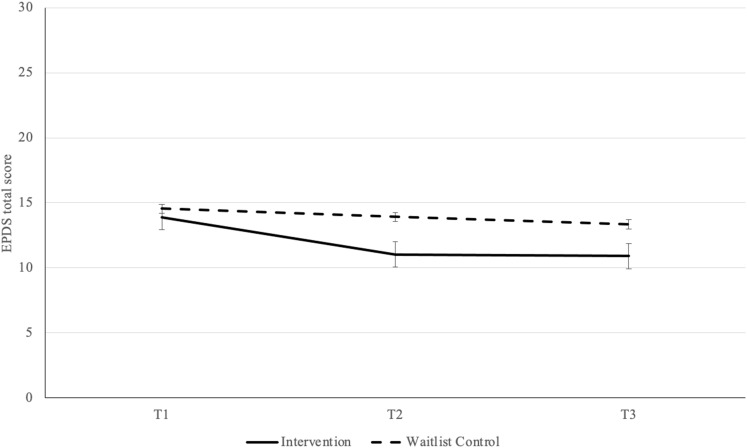
Postpartum Depression (EPDS) Scores by Time of Assessment and Group Reported as Mean ± SE. *Note*: Possible range of scores: 0 to 30, higher scores indicate more severe depression symptoms; EPDS Edinburgh Postnatal Depression Scale

**Fig. 4:**
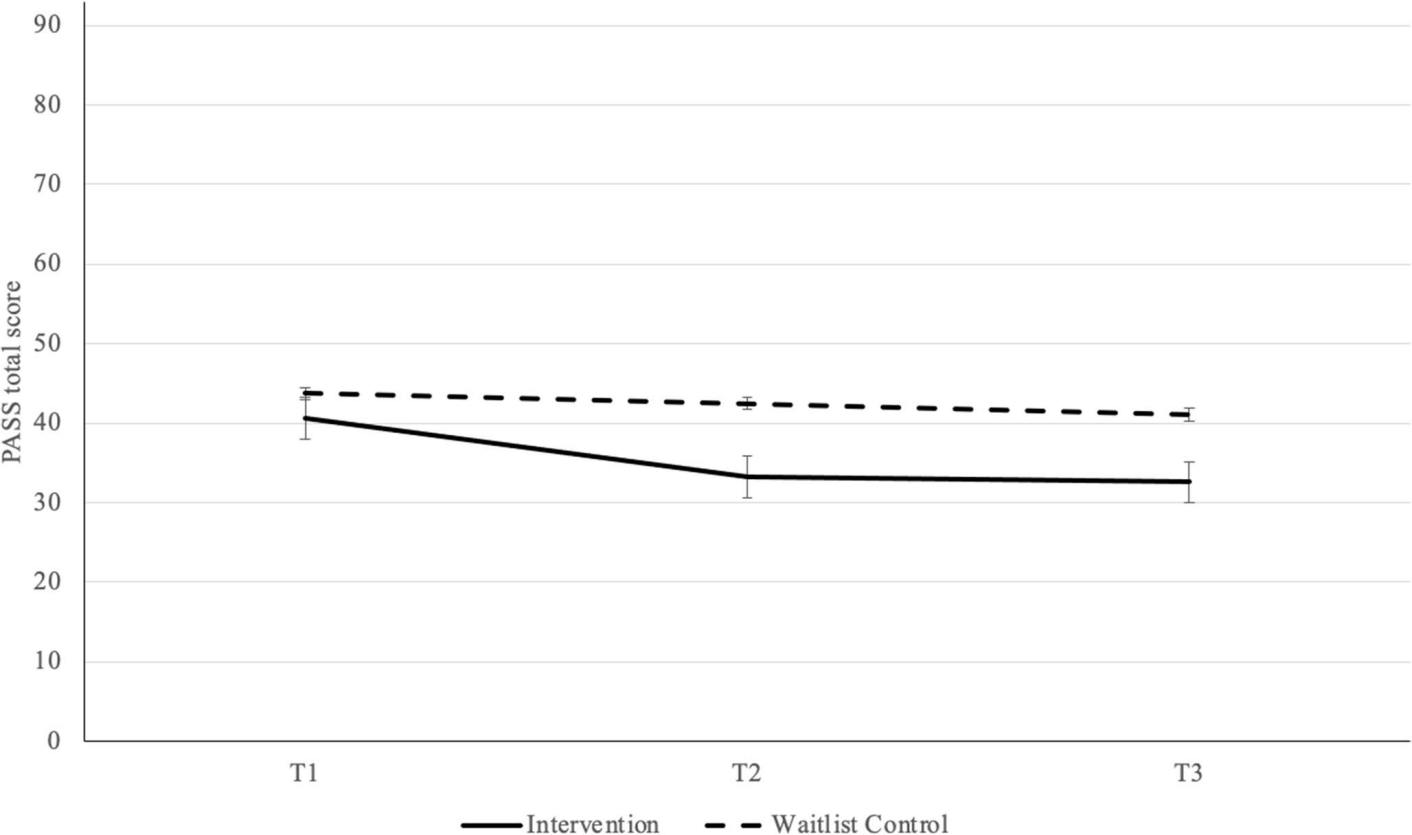
Postpartum Anxiety (PASS) Scores by Time of Assessment and Group Reported as Mean  ± SE. *Note*: Possible range of scores: 0 to 93, higher scores indicate more severe anxiety symptoms; PASS Perinatal Anxiety Screening Scale

Of 317 initially assessed, *N* = 103 met inclusion criteria and provided consent (Figs. 2, 3, 4). Following randomization, 101 completed T1 questionnaires, 95 completed T2 questionnaires, and 92 completed T3 questionnaires and were included in analyses for primary outcomes (intervention: *n* = 45, waitlist control: *n* = 47). From randomization to T3, 11 participants were lost to follow-up as they did not respond to reminders prompting questionnaire completion, resulting in a 10.7% attrition rate, which was lower than the anticipated 20% attrition rate. There were no additional reasons provided for study discontinuation, so we deemed these as “lost to follow-up”. All participants (*N* = 103) were included in demographic characteristic analyses. All participants who completed T1 questionnaires (*n* = 101) were included in analyses of baseline PPD/PPA symptoms and all intervention participants who completed T2 questionnaires (*n* = 47) were included in analyses for implementation outcomes.

Although participants of any gender were eligible for inclusion, all participants self-identified as cisgender women so we refer to our sample as “women” from here o. Analyses comparing demographic variables at T1 revealed a significant difference in age and education levels such that waitlist control participant were significantly older and more educated than intervention participants (Table 2). Waitlist control participants (*M* = 34.25, *SD* = 4.84) were significantly older than intervention participants (*M* = 31.60, *SD* = 5.12), *t*(2) = 2.71, *p* = .008. Waitlist control participants (*M* = 17.64, *SD* = 4.84) attended significantly more years of school compared to intervention participants (*M* = 16.06, *SD* = 3.16), *t*(97) = 2.62, *p* = .010, and had attained higher education levels, χ ^2^(6, *N* = 103) = 12.81, *p* = .046. To factor in the significant group differences in age and education at baseline, the interaction of time and group was also tested using ANCOVA with age and education as covariates. The results of ANCOVA produced the same pattern of significant interaction and time main effects for both primary outcomes and there were no significant interactions between time and covariates. Therefore, the originally planned ANOVA results are reported without age and education included.

### Primary outcome analyses

On the first primary outcome of PPD symptoms (EPDS total score), there was a significant main effect of time with scores decreasing over time, *F*(2,180) = 11.22, *p* < .001, partial η^2^ = 0.11, indicating a medium-to-large effect size, and a significant main effect of group favouring the intervention group *F*(1, 90) = 5.40, *p* = .022, partial η^2^ **= .**06, indicating a medium effect size (Table 3). Main effects were qualified by a significant interaction between group and time, *F*(2, 180) = 3.12, *p* = .046, partial η^2^ = 0.03, indicating a small-to-medium effect size (Adams and Conway 1966). Post-hoc pairwise-comparisons revealed a significant mean difference from T1 to T2 (*MD* = 2.85, *SE* = 0.65, *p* < .001), with the effect remaining significant at T3 (*MD* = 2.96, *SE* = 0.73, *p* < .001) for the intervention group only. For the waitlist control group, the mean difference from T1 to T2 did not reach significance (*MD* = 0.62, *SE* = 0.63, *p* = .327), and the effect remained non-significant at T3 (*MD* = 1.20, *SE* = 0.71, *p* = .096).

**Table 3:** Primary outcomes of postpartum depression and anxiety scores by time of assessment and group reported as mean ± SD

Outcome		Group	T1	T2	T3
PPD (EPDS)^1^		Waitlist Control	14.54 ± 4.37	13.91 ± 5.24	13.34 ± 5.44
		*Intervention	13.88 ± 4.86	11.02 ± 4.60	10.91 ± 4.59
PPA (PASS)^2^		Waitlist Control	43.74 ± 15.78	42.44 ± 15.67	41.08 ± 16.52
		*Intervention	40.63 ± 13.81	33.21 ± 14.64	32.57 ± 14.08

*Note* Statistics are reported for all participants who completed T3 questionnaires (intervention: *n* = 45; waitlist control: *n* = 47); Scale Range: ^1^0 to 30; ^2^0 to 93; For both outcomes, higher scores indicate more severe PPD/PPA symptoms; EPDS = Edinburgh Postnatal Depression Scale; PASS = Perinatal Anxiety Screening Scale; PPD = Postpartum Depression; PPA = Postpartum Anxiety

*Significant time by group interaction *p* < .05

On the co-primary outcome of PPA symptoms (PASS total score), there was a significant main effect of time with scores decreasing over time, *F*(2,178) = 11.12, *p* **<** .001, partial η^2^ = 0.11, indicating a medium-to-large effect size, and a significant main effect of group favouring the intervention group *F*(1, 89) = 5.92, *p* = .017, partial η^2^ = 0.06, indicating a medium effect size (Table 3). Main effects were qualified by a significant interaction between group and time, *F*(2, 178) = 3.79, *p* = .024, partial η^2^ = 0.04, indicating a small-to-medium effect size (Adams and Conway 1966). Post-hoc pairwise-comparison tests for the interaction effect revealed a significant mean difference from T1 to T2 (*MD* = 7.42, *SE* = 1.74, *p* < .001), with the effect remaining significant at T3 (*MD* = 8.07, *SE* = 1.89, *p* < .001) for the intervention group only. For the waitlist control group, the mean difference from T1 to T2 was non-significant (*MD* = 1.33, *SE* = 1.68, *p* = .430), and the effect remained non-significant at T3 (*MD* = 2.64, *SE* = 1.83, *p* = .148) .

### Clinical significance

For PPD, a chi-square test showed a significant relationship between group and PPD recovery, such that a greater proportion of participants recovered in the intervention group χ ^*2*^ (1, *n* = 63) = 4.58, *p* = .032 (Table 4). For PPA, although there were more that improved in the intervention group (*n* = 10, 27%) compared to the waitlist control group (*n* = 5, 11.9%) at T3, there was no statistically significant association between clinically significant PPA improvement and group, χ ^2^ (1, *n* = 79) = 2.92, *p* **=** .087 (Table 5).

**Table 4:** *Comparison of PPD recovery and group from T1 to T3 reported as N (%)*

Group	^a^EPDS decrease ≥ 4 points	EPDS change from > 12 to ≤ 12	^b^PPD Recovery	No ^b^PPD Recovery
Waitlist Control	9 (27.3)	13 (39.4)	7 (21.2)	26 (78.8)
*Intervention	17 (56.7)	15 (50.0)	14 (46.7)	16 (53.3)

Statistics are reported for participants who met clinical cut-off for PPD at baseline (EPDS > 12) (*n* = 63); EPDS = Edinburgh Postnatal Depression Scale; PPD = Postpartum Depression

^a^EPDS decrease of ≥ 4 points equivalent to Reliable Change Index ≥ 1.96, indicating the change in EPDS scores is likely to be due to a real change in the individual (95% confidence)

^b^PPD recovery indicated as both EPDS decrease ≥ 4 points and EPDS change from > 12 to ≤ 12 from T1 to T3.

*Significant relationship between group and PPD recovery, *p* < .05

**Table 5:** Comparison of clinically significant PPA improvement and group from T1 to T3 reported as N (%)

Group	PASS change from ≥ 26 to <26	No PASS change from ≥ 26 to <26
Waitlist Control	5 (11.9)	37 (88.1)
Intervention	10 (27.0)	27 (73.0)

Statistics are reported for participants who met the cut-off for PPA at baseline

(PASS ≥ 26) (*n* = 79); PASS = Perinatal Anxiety Screening Scale; PPA = Postpartum Anxiety

### Implementation outcome analyses

#### Patient global impression of change (PGIC)

Intervention participants (*n* = 47) provided ratings on three questions adapted from the PGIC. On the first question evaluating perceived improvement in PPD and/or PPA symptoms following the use of PostpartumCare.ca, the mean rating was 3.74, *SD* = 0.94. On the second question evaluating perceived improvements in overall quality of life, participants’ mean rating was 3.06, *SD* = 0.73. The final question rated overall satisfaction towards PostpartumCare.ca and the mean score was 5.56, *SD* = 2.05 (Table 6).

**Table 6:** *Descriptive statistics of website usability and satisfaction outcome variables by intervention participants at T2*

Outcome measure	*N*	M ± SD
^a^PGIC 1: PPD/PPA symptom improvement	47	3.74 ± 0.94
^b^PGIC 2: Quality of life	47	3.06 ± 0.73
^c^PGIC 3: Overall website satisfaction	46	5.56 ± 2.05
^d^SUS	47	67.29 ± 21.15
^e^PEMAT Understandability	45	85.23 ± 20.63
^e^PEMAT Actionability	45	84.07 ± 23.56
^f^UPWQI Technical Adequacy	47	5.17 ± 1.04
^f^UPWQI Content Quality	47	5.27 ± 1.32
^f^UPWQI Specific Content	47	4.97 ± 1.15
^f^UPWQI Appearance	47	5.31 ± 1.40

*Note* PGIC = Patient Global Impression of Change; PPA = Postpartum Anxiety; PPD = Postpartum Depression SUS = System Usability Scale; PEMAT = Patient Education Materials Assessment Tool; UPWQI = User-Perceived Web Quality Instrument

^a^Likert scale range from 1 (complete recovery) to 6 (deterioration)

^b^Likert scale range from 1 (great improvement) to 5 (deterioration)

^c^Scores represent percentage for understandability or actionability

^d^Scores range from 1 (completely disagree) to 7 (completely agree)

#### System usability scale (SUS)

Intervention participants (*n* = 47) rated the system usability of PostpartumCare.ca as about average overall (*M* = 67.29, *SD* = 21.15) (Table 6), given that an SUS score ≥ 68 indicates above average website usability (Bangor 2009).

#### Patient education materials assessment tool (PEMAT)

On average, intervention participants (*n* = 45) rated website understandability at 85.23%, *SD* = 20.63. Similarly, intervention women (*n* = 45) rated the website as 84.07% actionable on average, *SD* = 23.56 (Table 6).

#### User-perceived web quality index (UPWQI)

Intervention participants (*n* = 47) rated agreement with the website’s technical adequacy as *M* = 5.17, *SD* = 1.04. Agreement with PostpartumCare’s content quality was rated as *M* = 5.27, *SD* = 1.32, and specific content was rated as *M* = 4.97, *SD* = 1.15. Finally, agreement with website appearance was rated as *M* = 5.31, *SD* = 1.40 (Table 6).

#### Website metrics

Website metrics for the intervention group (*n* = 47) were collected according to website section (Education, Supports, Resources, Mood/Anxiety Tracker) at T2 (see Table 7). Of the 47 participants who completed T2 questionnaires, 4 did not access the website during the study period but were still included in analyses for website metrics.

**Table 7:** Website metrics of intervention participants by website section

Website section	Average page visits per participant	Average time per session (minutes)	Total page views	Most visited outsources	
*Education*	1.06	48	56	→ Perinatal Services BC: health professionals page → Perinatal Services BC: EPDS questionnaire	
*Supports*	0.93	37	49	→ PsychCentral: self-care info blog → Pacific Postpartum Support Society: self-care questionnaire	
*Resources*	0.23	106	12	→ Villageofmoms.com → Postpartumdads.org	
*Mood/Anxiety Tracker*	1.02	33	82	→ PsychCentral: self-care tips blog	

*Note n =* 47; BC = British Columbia; EPDS = Edinburgh Postnatal Depression Scale

In total, the website was accessed 113 times, an average of 2.40 (*SD =* 1.90) times per participant. The Education section was visited an average of 1.06 times per participant, for an average time of 48 min per session and received 56 total page views. The Supports section was visited an average of 0.93 times per participant for an average time of 37 min per session and received 49 total page views. The Resources section was visited an average of 0.23 times per participant for an average time of 106 min per session and received 12 total page views. The Mood/Anxiety Tracker was visited an average of 1.02 times per participant for an average time of 33 min and received 82 total page views. The number of Mood/Anxiety Tracker entries per participant ranged from 0 to 6 total entries within the four-week study period. The most viewed outsources per section are reported in Table 7.

## Discussion

### Principal findings

Results showed that engagement with PostpartumCare.ca significantly reduced symptoms of PPD and PPA after four weeks in the intervention group only, with improvements maintained after a two-week follow-up. These improvements were associated with small-to-medium effect sizes, and clinical significance outcomes showed intervention participants to be more likely to recover from PPD. Website usability and satisfaction were rated favourably overall by intervention participants. Global patient impression of change scores indicated a small-to-moderate perceived improvement in depression and anxiety symptoms, a small improvement in perceived overall quality of life, and slightly above average overall satisfaction with the intervention with scores falling in the middle range. System usability was rated as average overall, understandability and actionability were highly rated, and women generally agreed with the website’s technical adequacy, content quality, specific content, and appearance.

Website metrics also provided some insights as to how PostpartumCare.ca may be used. Intervention participants viewed most sections about once on average and spent anywhere from 33 to 106 min on each section per session, suggesting that website visits may be somewhat infrequent, but of considerable duration.

### Web-enabled psychoeducational resource can improve symptoms of PPD and PPA

These findings build upon previous research indicating that psychoeducational interventions can be effective for reducing symptoms of depression and anxiety in various patient populations and may be a valuable first-line intervention for those experiencing psychological distress (Al-Alawi et al. 2022; Chan et al. 2019; Donker et al. 2009; Williams et al. 2014). Further, our study found evidence of the effectiveness of a psychoeducational resource for mental health delivered via eHealth and applied to a sample of postpartum women.

Although psychoeducational interventions are known to serve purposes such as education and prevention, research also suggests that psychoeducation can be effective for reducing symptoms of psychological disorders including depression and anxiety and are generally less expensive, less time-consuming, more easily administered, and potentially more accessible than other psychotherapeutic interventions (Donker et al. 2009). This may be particularly relevant for those facing postpartum mental health challenges considering the added time and financial constraints associated with postpartum and early parenthood (Button et al. 2017; Jones 2019).

Previous literature investigating eHealth interventions for the prevention and treatment of PPD and PPA have largely focused on interventions that incorporated a direct psychotherapeutic component, often centered on CBT or Behavioural Activation principles (Saddichha et al. 2014). However, the body of literature investigating the effects of eHealth-delivered psychoeducational resources without direct psychotherapeutic components for reducing postpartum mental illness is limited, and existing studies have shown mixed results. One study found a reduction in PPD symptoms following the use of a smartphone-based psychoeducational intervention in first time mothers, but reductions in anxiety symptoms were not significant (Chan et al. 2019). Another study found no significant difference in PPD symptoms between intervention and control groups following the use of a psychoeducational mobile-health application, and PPA was not evaluated (Shorey et al. 2017). Our findings suggest that psychoeducational content delivered online can be effective for reducing symptoms of PPD and PPA. Because they are informational in nature, the specific content delivered may be key in influencing the effectiveness of an intervention (Donker et al. 2009). Of note, both interventions from Chan et al. (2019) and Shorey et al. (2017) provided information related to general parenting rather than tailored to perinatal mental health disorders. The effectiveness of the current intervention may be attributed to women responding favourably to psychoeducational material specific to their underlying mental health disorder(s). Future research should therefore continue to investigate the effectiveness of web-enabled psychoeducational tools for postpartum mental health to further understand what types of information provide the most benefit to those in need of support.

### User perceptions of postpartumCare.ca

Following use of PostpartumCare.ca, participants on average perceived small-to-moderate improvements in depression and anxiety symptoms and a small improvement in overall quality of life. User feedback was positive regarding the website’s actionability and understandability, and towards specific website aspects such as technical adequacy, content quality, and appearance. These findings are in agreement with previous results from a pilot study of a prototype of the current intervention where the website’s layout, language, and content were found to be generally easy to understand, clear, trustworthy, and helpful by nearly all participants (Siddhpuria et al. 2022). However, on measures that assessed overall satisfaction and system usability, our results fell in the middle range, around average. This finding differs slightly from Siddhpuria et al. (2022), where most participants were either somewhat or very satisfied with the website protypes. A possible explanation for the more modest satisfaction ratings in the current study may be reflected in the website metrics of participants, as some key aspects of the site may not have been accessed to their fullest potential. Most pages were viewed an average of one time per participant. Because of this infrequent usage pattern, participants may not have been able to fully utilize website features intended for repeated usage such as the Mood/Anxiety Tracker, which was intended to allow participants to create entries on a more regular basis to track mood and anxiety symptoms over time. However, this study did not collect direct participant feedback related to usability and satisfaction of individual website sections (i.e., Education, Resources, Supports, Mood/Anxiety Tracker) and therefore, it is difficult to confirm how satisfaction ratings might have been related to participant experiences with individual website sections/features. As such, specific reasons as to why features such as the Mood/Anxiety Tracker were not utilized as frequently as initially intended are unclear. Despite this, when considering all measures of website usability and satisfaction together, the website was reviewed favorably overall by participants in this study, evidencing its potential usefulness for women.

### Clinical implications

Women with clinical levels of PPD who used PostpartumCare.ca were more likely than controls to recover from PPD. This finding, along with the statistically significant improvements in symptoms, suggest our web-enabled resource may be helpful for the alleviation of clinical PPD symptoms. These findings, though, are difficult to confirm without the use of gold-standard methods for evaluating clinical PPD/PPA diagnosis (i.e., diagnostic interviews based on DSM-5 criteria). Therefore, future research employing gold-standard diagnostic methods is suggested to better understand the effect of a web-enabled psychoeducational tool on clinical PPD and PPA symptom recovery.

### Limitations and strengths

This study does have some limitations. First, the demographic homogeneity of our sample (e.g., all cisgender women, mostly White, educated, heterosexual, with relatively high income) may limit the generalizability of our findings to the greater BC population. Second, this study lacks longer-term follow-ups beyond two weeks after engaging with PostpartumCare.ca, thus limiting our understanding of the potential long-term impact of the intervention on symptoms of PPD/PPA. Finally, this study did not collect qualitative participant feedback on website utilization (i.e., investigating why participants spent more/less time on certain pages). Therefore, specific reasons as to why certain sections were utilized more/less by each participant are unclear.

Despite these limitations, our study had notable strengths. To our knowledge this is the first study to evaluate a web-enabled psychoeducational intervention specific to the needs of women in BC affected by postpartum mental illness and that was developed according to principles of user-centered design (Canadian Institutes of Health Research 2011; Still and Crane 2017; User-Centered Design Basics 2017). Few published studies evaluating digital postpartum mental health resources have incorporated the same degree of iterative patient engagement, utilizing user feedback at each stage of intervention development and assessment (Chan et al. 2019; Fealy et al. 2019; Haga et al. 2013; O’Mahen et al. 2014; Pugh et al. 2016; Shorey et al. 2017). Considering the increasing reliance on web-enabled resources in many health domains, patient engagement should be prioritized given its feasibility and benefits for intended populations. Second, this study had relatively low attrition compared to similar studies in the field (Chan et al. 2019; Dennis et al. 2020). Finally, our intervention addressed both PPD and PPA, setting it apart from many existing resources that target depression only (Loughnan et al. 2019).

### Conclusions and future directions

Our findings suggest that four-week usage of a web-enabled psychoeducational intervention for postpartum mental health is effective for reducing symptoms of PPD and PPA among women in BC. As a follow-up to the current trial, a future knowledge translation phase will seek to develop mobilization strategies for implementing PostpartumCare.ca as a practical tool for women in BC.
